# Buprenorphine for High-dose Tramadol Dependence: A Case Report of Successful Outpatient Treatment

**DOI:** 10.5811/cpcem.2021.12.54602

**Published:** 2022-01-28

**Authors:** Leslie Mukau, Kadia Wormley, Christian Tomaszewski, Bushra Ahmad, Rais Vohra, Andrew A. Herring

**Affiliations:** * University of California, San Diego, Department of Emergency Medicine, San Diego, California; † El Centro Regional Medical Center, Department of Emergency Medicine, El Centro, California; ‡ Highland Hospital – Alameda Health System, Department of Emergency Medicine, Oakland, California; § Imperial County Behavioral Health Services, Division of Substance Use Disorder Treatment Program, El Centro, California; ¶ University of California, San Francisco-Fresno, Department of Emergency Medicine, Fresno, California; || University of California, San Francisco, Department of Emergency Medicine, San Francisco, California

**Keywords:** case report, tramadol addiction, serotonin-norepinephrine reuptake inhibitor (SNRI) withdrawal symptoms, buprenorphine induction

## Abstract

**Introduction:**

During the coronavirus disease 2019 pandemic caused by the severe acute respiratory syndrome coronavirus 2, deaths from opiate drug overdoses reached their highest recorded annual levels in 2020. Medication-assisted treatment for opiate use disorder has demonstrated efficacy in reducing opiate overdoses and all-cause mortality and improving multiple other patient-centered outcomes. Treatment of tramadol dependence in particular poses unique challenges due to its combined action as opioid agonist and serotonin-norepinephrine reuptake inhibitor. Tramadol puts patients with dependence at risk for atypical withdrawal syndromes when attempting to reduce use. Little evidence is available to guide treatment of tramadol dependence.

**Case Report:**

We present a case of high-dose tramadol addiction that began with misuse of medically prescribed tramadol for treatment of musculoskeletal back pain. The patient’s use reached oral consumption of 5000–6000 milligrams of illicit tramadol daily. She complained of common complications of tramadol use disorder including memory impairment, excessive sedation, and tramadol-induced seizures. The patient was referred to the emergency department in a withdrawal crisis seeking treatment where she was successfully managed with buprenorphine and phenobarbital and then linked to ongoing outpatient treatment.

**Conclusion:**

Our report adds to the limited guidance currently available on the acute management of tramadol withdrawal and treatment of tramadol use disorder. Our case suggests the initiation of high-dose buprenorphine may be an effective and feasible option for emergency clinicians.

## INTRODUCTION

In the midst of the coronavirus disease 2019 pandemic caused by the severe acute respiratory syndrome coronavirus 2, drug overdose deaths rose nearly 30% to a record 93,000 in 2020, representing the most drug overdose deaths in a year, the most deaths from opioid overdoses, and the most overdose deaths from synthetic opioids.^1^ Treatment of opioid use disorder with buprenorphine or methadone has been shown to decrease opioid overdose, reduce all-cause mortality, improve quality of life, decrease human immunodeficiency virus/hepatitis C transmission, and reduce drug cravings and criminality.^2^

Tramadol is a centrally acting opioid agonist and serotonin/norepinephrine reuptake inhibitor (SNRI) used for the management of moderate to severe pain in adults. Tramadol differs from other traditional opioid medications in that it doesn’t just act as a μ-opioid agonist, but also affects monoamines by modulating the effects of neurotransmitters involved in the modulation of pain such as serotonin and norepinephrine, which activate descending pain inhibitory pathways.^3^ Unlike other opioid medications, tramadol use, especially at sustained high doses also carries a risk of seizure and serotonin syndrome, especially if used with other serotonergic medications.^4^ Unfortunately, there is little in the literature to guide emergency treatment of tramadol addiction.

Although attempts to treat tramadol withdrawal with buprenorphine have been published, this is the first case of high-dose tramadol addiction and dependence successfully managed with buprenorphine in an emergency department (ED) setting. Given the increased interest and use of ED-initiated buprenorphine we believe cases like this one could be a useful guide for other clinicians confronted by similar cases.

## CASE REPORT

A 29-year-old Latina female with a past medical history of post-traumatic stress disorder (PTSD), depression, and anxiety self-referred to a behavioral health center seeking treatment for severe tramadol use disorder. She had a remote history of marijuana use, without other recreational drug or alcohol use. She had no history of any other opioid use, apart from tramadol. At age 24 she first began taking tramadol 50 milligrams (mg) daily as prescribed by her primary care physician for treatment of back pain but continued use after this pain had resolved. She began crossing the border into Mexico to purchase tramadol in increasing quantities and slowly increased her dose to approximately 5000–6000 mg daily, costing her $200 US dollars monthly.

Complications of her tramadol use included memory impairment, excessive sedation, and tramadol-induced seizures, occurring about every two weeks. Prior to presentation for care, she had independently tried numerous times to quit by tapering but was limited by intolerable withdrawal symptoms, never dropping usage below 4000 mg daily. At time of presentation, she had not yet participated in any formal detoxification program. Withdrawal symptoms began two hours after her last use and included anxiety, restlessness, diaphoresis, and arthralgias. During this time, she was concomitantly experiencing hopelessness and passive suicidality in the setting of untreated depression, anxiety, and PTSD from childhood sexual, physical, and emotional abuse. Her family history was notable for active substance dependence in multiple members, including a sister who had recently died from a heroin overdose four months prior to presentation. Her mother had a history of methamphetamine abuse, and her brother was actively abusing multiple illicit drugs including fentanyl.

**CPC-EM Capsule**

What do we already know about this clinical entity?

*Opioid use disorder (OUD) is an intensifying national epidemic due to many factors including overuse of prescription pain medication and relative lack of resources for those seeking OUD recovery*.

What makes this presentation of disease reportable?

*Tramadol is widely prescribed and can cause toxicity due to both opioid and serotonin effects. Opioid withdrawal symptoms from tramadol may be managed with buprenorphine*.

What is the major learning point?

*Rapid identification of tramadol use disorder and tramadol withdrawal with urgent induction of buprenorphine can help patients avoid the complications related to tramadol addiction*.

How might this improve emergency medicine practice?

*Clinicians can help prevent overdose, complications, and deaths from tramadol overuse by identifying OUD in emergency department patients and offering medication assisted treatment with buprenorphine*.

Given tramadol’s combined action as a mu-receptor agonist and SNRI, the patient was at risk for an atypical opioid withdrawal syndrome. For this reason, inpatient detoxification with tramadol tapering and buprenorphine induction was preferred. Ultimately, given limitations of local resources and in consultation with addiction specialists, a plan was made to coordinate outpatient buprenorphine induction from the ED. Seven days following initial presentation to the behavioral health facility the patient was asked to go to the ED but did not and decided to taper the tramadol dose herself. She went down to 4000 mg of tramadol per day but started having withdrawal symptoms and went back up to 5000–6000 mg a day. Three days later she finally showed up for her first ED visit.

At her first outpatient induction attempt, she presented to the ED and was given sublingual buprenorphine 8 mg with phenobarbital 200 mg added to prevent withdrawal seizures. She was discharged home on buprenorphine/naloxone 8/2 mg twice a day with instructions to return the next day for follow-up. On the night of discharge, she noted significant withdrawal symptoms, reported difficulty with sleep and anxiety, and ultimately resumed tramadol use. The patient never filled her prescriptions. She received additional counseling regarding her available treatment options: slowly tapering use vs medication-assisted treatment.

On one of her trips from Mexico the patient was apprehended for illegal drug possession, and all her tramadol pills were confiscated. She returned to the ED eight days later after her initial visit. Before coming to the ED, the patient had taken buprenorphine/naloxone (8/2) mg. After examination she was given an additional 8 mg buprenorphine. About an hour later she was feeling slightly better but still having some residual withdrawal symptoms. She was given another 8 mg of buprenorphine. She felt much better and was discharged after spending slightly less than four hours in the ED.

She was discharged with a prescription for buprenorphine/naloxone 16/4 mg twice a day. Venlafaxine, a SNRI, was concomitantly prescribed to forestall possible SNRI withdrawal symptoms. Ten days post induction, she was still taking prescribed buprenorphine/naloxone at the same dose and was not having withdrawal symptoms, drug cravings or using tramadol. She had not yet started taking venlafaxine. Almost a year out after induction, she reported stable abstinence from tramadol with buprenorphine/naloxone 16/4 mg twice a day. She had also started treatment for depression and anxiety with buspirone 10 mg and sertraline 150 mg once daily.

## DISCUSSION

We present a complicated case of high-dose tramadol addiction and dependence successfully treated with high-dose buprenorphine induction and high-dose buprenorphine maintenance initiated in the ED setting. Previous case studies have shown some success with transitioning tramadol-dependent patients to buprenorphine. Using a residential inpatient treatment facility, a patient with a dependence of 1400 mg of tramadol a day was transitioned successfully over 28 days to stable treatment with buprenorphine 8 mg/naloxone 2 mg orally daily.^5^ The biggest hindrance was complications with antidepressant discontinuation syndrome, which was due to tramadol’s serotoninergic activity.^6^ Hence, we offered the patient a prescription for venlafaxine, which she did not fill, in addition to the buprenorphine.

After hydrocodone and oxycodone, tramadol is the third highest used and misused opioid per data from the Drug Abuse Warning Network, a nationwide public health surveillance system that improves ED monitoring of substance use crises, including those related to opioids, with over a million cases of misuse reported annually.^7^ Tramadol abuse accounts for over 20,000 ED visits annually.^8^ The effect of rescheduling hydrocodone from schedule III to II in 2012 has been associated with an increase in tramadol prescribing based on data available in four states.^9^ In addition to opioid dependence and adverse effects, such as seizures and serotonergic syndrome associated with tramadol, its use naively for post-surgical pain is associated with an increased risk of prolonged opioid use when compared to other short-acting opioids.^10^ Its use has also been associated with increased all-cause mortality compared to non-opioid pain medications, suggesting it is no safer than traditional opioids.^11^ Therefore, in addition to preventing opioid dependence, it behooves clinicians to wean patients off tramadol, especially when they are using excessively high levels, since toxicity of this drug is high.

In the case of our patient there were concerns for unpleasant SNRI discontinuation syndrome and withdrawal seizures due to tramadol dose tapering, but we managed without inpatient admission. Since tramadol is also an SNRI we were uncertain whether we should be concerned about SNRI withdrawal syndrome and whether the patient should also have been concomitantly started on an antidepressant in addition to buprenorphine. We prescribed an antidepressant, venlafaxine, but she did not take it. The patient reported that initial attempts with lower doses of buprenorphine did not adequately treat withdrawal symptoms and craving. High-dose buprenorphine appears to have been successful for this patient.

Physical dependence on tramadol can occur at doses as low as 200 mg/day.^12^ In addition to the usual opioid withdrawal symptoms tramadol may have atypical opioid withdrawal syndrome symptoms that may include unusual extremity sensory experiences including numbness and prickling, hallucinations, confusion, intense paranoia, high anxiety and panic attacks, and disorientation and depersonalization.^13^ Although these atypical symptoms may not be generally life-threatening, they may be uncomfortable or put the individual in dangerous situations or at high risk of making bad decisions.

Literature is sparse regarding how to treat such individuals short of an inpatient, medically supervised detox center. Herring et al have recently shown that high-dose buprenorphine (high-dose induction dose defined as greater than 12 mg) is both efficacious and safe in treating patients with opioid use disorder in the ED.^14^ Extended-release (ER) tramadol has been shown to be as effective as buprenorphine for treating opioid withdrawal in two randomized controlled trials. Doses up to 600 mg/day of tramadol ER were used successfully in one randomized controlled trial, but the drug was quickly tapered over one week during their residential treatment.^15^ Another study showed that buprenorphine results in lower withdrawal symptoms within two to three days of detoxification vs tramadol.^16^ The downside in that trial was that three patients (10%) sustained seizures, limiting tramadol’s use for severe opioid dependence long term. Therefore, substituting high-dose buprenorphine for opioids, including tramadol, may be more efficacious for induction and sustainability in patients with high-dose opioid dependence, particularly those who are trying to end tramadol dependence.

## CONCLUSION

Little has been written about specific treatment for patients with tramadol use disorder. This case illustrates that buprenorphine induction and maintenance without concomitant use of an SNRI agent may be all that is needed in high-dose tramadol detoxification and or treatment of withdrawal symptoms in an outpatient setting.
